# Subsyndromal states in bipolar disorder

**DOI:** 10.4103/0019-5545.74314

**Published:** 2010

**Authors:** Dushad Ram, Daya Ram

**Affiliations:** Department of Psychiatry, JSS Medical College Hospital, Mysore, India; 1 Central Institute of Psychiatry, Ranchi, Jharkhand, India

**Keywords:** Bipolar disorder, subsyndromal symptoms

## Abstract

**Background:**

Despite adequate treatment, patients with bipolar disorder suffer from subsyndromal symptoms. This study has been done to see the association of subsyndromal symptoms with age of onset, duration of illness, duration of episodes, and number of episodes.

**Aims:**

To know the prevalence of subsyndromal symptoms and their relationship with age of onset, total duration of illness, number of episodes, and duration of episodes in patients with bipolar disorder in remission.

**Materials and Methods:**

The study was cross sectional and hospital based. One hundred patients, aged between 18 and 65 years, diagnosed as bipolar disorder according to Research Diagnostic Criteria of ICD-10, and with good compliance with the prophylactic medications, score of ≤5 on Beck’s Mania Rating Scale (BMRS) and score ≤8 on Hamilton Depression Rating Scale (HDRS) were recruited by the purposive sampling method. Descriptive statistics were used to describe various sample characteristics. Group differences for categorical variables were examined with the chi-square test, whereas an independent *‘t’* test was used for continuous variables.

**Results:**

The most common manic symptom was a decrease in sleep (49%), followed by an increase in verbal activity (39%), hostility (37%), and increase in motor activity (33.33%). The subsyndromal manic group had a lower age of onset (58.8%), males (82.4%), unemployed (23.5%), educated (80.4%). There was no significant difference between with and without subsyndromal mania groups with respect to age, age of onset, duration of illness, number of episode, and average duration of episode.

**Conclusion:**

Subsyndromal manic symptoms are prevalent and have no relationship with current age, age of onset of illness, duration of illness, number of episode, and average duration of illness in patient with bipolar disorder in remission.

## INTRODUCTION

The specification of diagnostic algorithms and cut off scores has led to the new controversy as to whether the sub-threshold/subsyndromal symptoms are a clinical problem at all, or separate phenomenon in their own right, or a minor form of major disorder, or methodological artifact created by possibly invalid definition of algorithms and threshold in the present classification systems. Such symptoms are often seen in patients with bipolar disorder and is contrary to the traditional view of remission as cardinal feature.[1] Studies revealed prevalence of subsyndromal symptoms in bipolar disorder ranging from 13 to 70%.[2–5] However, some studies have failed to detect such symptoms.[6] There are few studies done on subsyndromal mania in bipolar disorder in India and tended to focus more on subsyndromal depression. The current study was under taken to find out sociodemographic correlates of subsyndromal manic symptoms in patients with bipolar disorder.

## MATERIALS AND METHODS

The study was cross sectional and hospital based conducted at Central Institute of Psychiatry, Ranchi. It has got a wide catchments area that includes Bihar, Uttar Pradesh, West Bengal, Himachal Pradesh, Orissa, Madhya Pradesh, Chhattisgarh, and neighboring countries like Nepal, Bhutan, and Bangladesh.

A total of 100 remitted bipolar patient living in the community after the treatment of the acute phase of illness and currently only on prophylactic mood stabilizer, with regular follow up, were recruited for this study by the purposive sampling method provided they fulfilled the inclusion and exclusion criteria. Inclusion criteria were both male and female patients, aged between 18 and 65 years, with a diagnosis of bipolar diagnosis according to RDC of ICD 10 criteria,[7] and on prophylactic mood stabilizer with a good compliance. Exclusion criteria were significant medical illness or co-morbidity, presence of any other psychiatric disorders, last episode mixed, score 5 or more on BMRS,[8] and score 8 or more on HDRS.[9] Information was collected from the patient him/herself, key person/relative accompanying the patient, case record, and treating psychiatrist.

Tool for assessment used in this study are as follows.

1. *Socio-demographic and clinical proforma:* This consist of patient details including age, sex, occupation, education, age of onset of illness, present history of illness, past history of psychiatric and medical illness, personal history, duration of episode, dose of mood stabilizer, compliance, physical, and mental status examinations and diagnosis.
2. *Beck’s mania rating scale.*[8] It consists of 11 items. A total score 0 to 4 indicates no syndrome. A score of 5 or more indicates a manic syndrome. In order to make the diagnosis of subsyndromal mania this scale was modified as follows: score of 0 = no sub syndrome, score 1-4 = sub syndrome is present.
3. *Hamilton depression rating scale.*[9] The enlarged Hamilton depression rating scale consists of the 17 original Hamilton items and 5 Bech-Refaelsen items given in the depression scale. A total score of 0-7 indicates that there is no depression. A score of 8 or more shows that depression is present. In order to make the diagnosis of subsyndromal depression, this scale was modified as follows: score of 0 = no syndrome and score of 1-7= sub syndrome present.
4. *Udvalg for kliniske undersogelser scale for side effect.*[10] This is an observer scale for assessment of side effects of drugs. The interview was supplemented by clinical observation and information obtained from relatives and/or case records. If there was discrepancy between patient’s symptoms and clinical signs, clinical observation was given precedence. Each item was rated on 4 item scale (0, 1, 2, and 3). ‘0’ indicating ‘not or doubtfully present’ and referred as normal.

### Statistical analysis

The data were analyzed by using statistical package for social science-version 10.1 (SPSS-10). For categorical data, frequency, percentage and chi-square tests, and for continuous data, mean standard deviation, and *t* test were used.

## RESULT

A majority of the patients had age of onset before 30 years (54%), male (76%), married (79%), living in joint family (71%), belonged rural area (59%), and educated between middle to intermediate level (74%) [Table 1].

**Table 1:** Sociodemographic variables

Variables	With subsyndromal mania N=51		Without subsyndromal mania N=46	
	n	%	n	%
Age at onset				
<30	30	58.8	22	47.8
>30	21	41.2	24	52.8
Sex				
Male	42	82.4	32	69.6
Female	9	17.6	14	30.4
Occupation				
Unemployed	12	23.5	3	6.9
Service	7	13.7	5	10.9
Farmer	9	17.6	11	23.9
Housewife	11	21.6	8	17.4
Student	6	11.8	8	17.4
Business	6	11.8	11	23.9
Education				
Uneducated	1	1.9	9	19.6
Primary	7	13.7	1	2.2
M-I level	41	80.4	30	65.2
Gr-PGr	2	3.9	6	13.0
Residence				
Rural	29	56.9	28	60.9
Urban	22	43.1	18	39.1
Marriage				
Unmarried	14	27.5	6	13.1
Married	37	72.5	32	69.6
SES				
Low	30	58.8	20	43.5
Middle	21	41.2	26	56.5
Type of family				
Nuclear	14	27.5	14	30.4
Joint	37	72.5	32	69.6
Family history				
Absent	26	50.9	24	52.2
Present	25	49.1	22	47.8

The most common manic symptom was decrease in sleep (49%). Other symptoms were an increase in verbal activity (39%), hostility (37%), increase motor activity (33.33%), elevated mood (27.45%), inflated self-esteem (15.7%), and flight of idea (5.9%). Common side effects seen were tremor (25%) difficulty in concentration (7%) [Table 2].

**Table 2:** Diagnosis of subsyndromal mania

Variables	Patient Group (N=100)	
	n	%
Subsyndromal mania (score 1-4 on BMRS)	51	51
No subsyndromal mania (score 0 on BMRS)	49	49

The data analysis showed [Tables 1 and 2] that in the subsyndromal mania group a large number had lower age of onset (58.8%), male (82.4%), unemployed (23.5%), educated (80.4%), unmarried, and from lower socio-economic status as compared to the no-subsyndromal mania group. The no-subsyndromal mania groups had sizable percentages of person doing business (23.9%), uneducated (19.6%), and were married (86.9%) [Table 3].

**Table 3:** Correlation of subsyndromal symptoms

Variables	With subsyndromal mania N=51		Without subsyndromal mania N=46			
	M	SD	M	SD	t	*P*
Age	31.4	+ 9.49	31.7	+ 11.18	0.133	NS
Age at onset	27.7	+ 9.13	24.0	+ 9.43	0.385	NS
Total duration	6.7	+ 4.17	7.9	+ 6.61	1.030	NS
No. of episode	3.0	+ 1.26	2.0	+ 1.12	1.258	NS
Duration episode	2.2	+ 1.26	2.0	+ 1.12	0.942	NS

The result showed no significant difference between the two groups in respect to current age, age at onset, duration of illness, number of episodes, and average duration of episode.

## DISCUSSION

Socio-demographic and clinical features of this study broadly coincide with the finding of the literature published from India.[11–13] Sex difference observed in this study may be because males suffer more,[14–16] and have greater severity,[17,18] with tendency for aggressive behavior,[5] and utilize mental health services more.[18] In Indian setup, sex differences could also be due to socio-cultural factors and stigma associated with females availing mental health service. The family’s occupational and economic status observed in this study is in keeping with traditional Indian socio-cultural agrarian background. A greater number of educated patients reflect increased awareness and better identification of the illness, and thus seeking help. Interestingly our study found that 45% of patients had a family history of affective illness either in first and/or second-degree relative/s. This finding indicates the familial nature of bipolar disorder,[19] a more severe form,[20] associated with multiple episodes,[21] and early age at onset.

### Frequency of depressive and manic symptoms

Most common manic symptoms observed were decreased, increased verbal activity, hostility, increased motor activity, and elevated mood which are comparable to findings observed in western countries.[2,22,23]

Common side effects observed in this study were tremor, difficulty in concentration, sedation, and orthostatic giddiness. These are well-known side effect of mood stabilizer used for prophylaxis.[24]

Out of all the patients, 51% had sub-syndromal manic symptoms in this study. Though few studies have failed to show any sub-syndromal symptoms in bipolar disorder in remission,[6] other studies have found such symptoms in up to 70% of the patients.[4] Such discrepancy may be related to methodology and to the scales used for assessment. Keller *et al*.[25] observed that these symptoms were due to inadequate doses of prophylactic medication (Lithium), while Goodnick *et al*.[26] found them to be independent of prophylactic medication.

Khanna *et al*.[27] found that in India recurrent manic pattern is more prevalent than typical bipolarity. Ram and Mathew[12] (1993) found that their patients had positive family history in up to 50% of cases, 53.3% having severe episode(s), and relatively unfavorable treatment response and with a tendency to have multiple episodes.

The patients with sub-syndrome tend to have early age at onset. They are more often male, unemployed, educated, unmarried, urban based, and from a joint family and lower socioeconomic status, and with a family history of affective illness. These variables are often reported to associate unfavorable outcome in bipolar disorder. Baur *et al*.[28,29] (2009) found that subsyndromal symptoms may preclude full-time responsibilities outside the home and suggested to incorporate these symptoms as outcome measure.

### Group difference between patients with and without sub-syndromal

This study revealed no significant difference between those with and without sub-syndromal status, though there were relatively more patients with early age of onset, total duration in the sub-syndromal manic group. Harrow *et al*,[25] found such a relation after 6 months of an index episode. Exclusion of patient with comorbid disorder, that is known to be associated with subsyndrome,[30] may also result in such observations. The heterogeneous nature of the illness and combined effect of multiple factors may be associated with such symptoms.

On the basis of the above findings following conclusions can be drawn that sub-syndromal symptoms are common in patients with bipolar disorder and does not seem to be related to current age, age at onset, illness duration, number of episode and duration of illness.[31] This result may be more relevant to patient attending psychiatric hospital at a tertiary centre for follow up probably after treatment of severe episode of illness.

The cross sectional study design, the exclusion of premorbid temperament / personalities and psychosocial factor as part of assessment are some of the limitations of this study.
